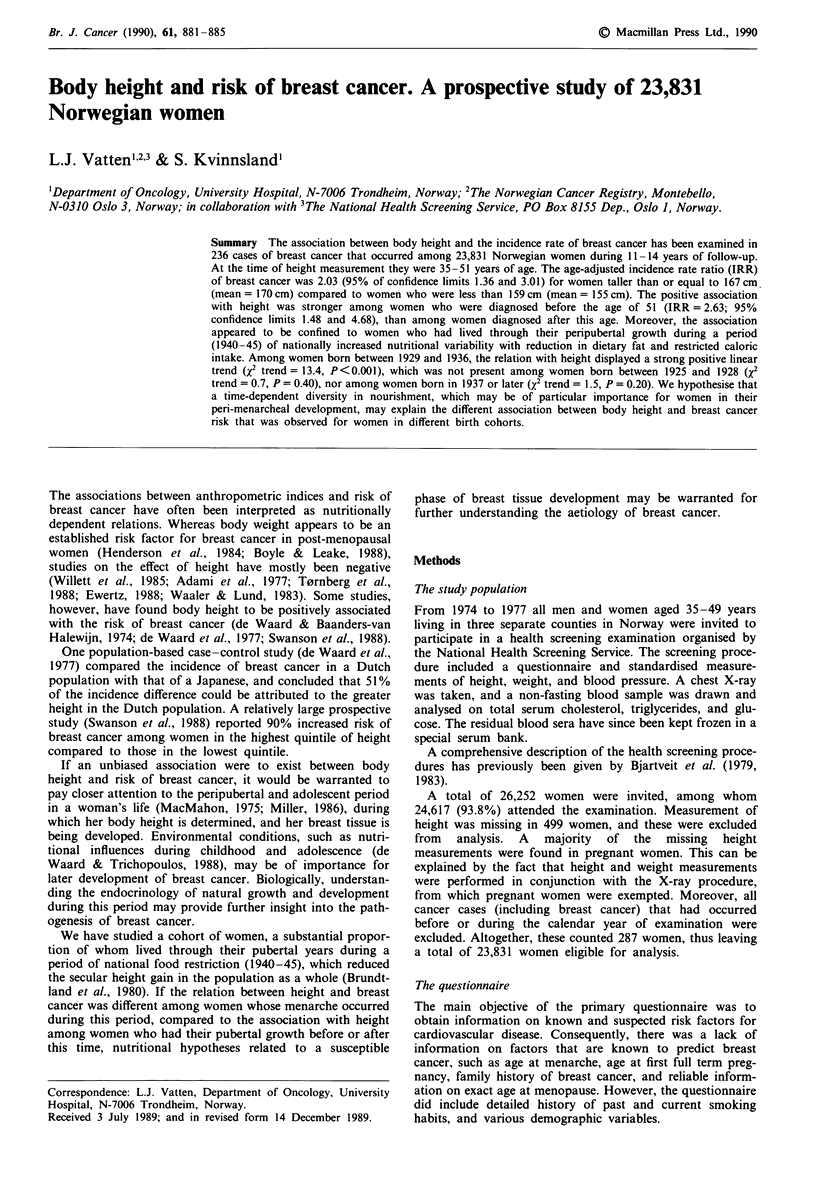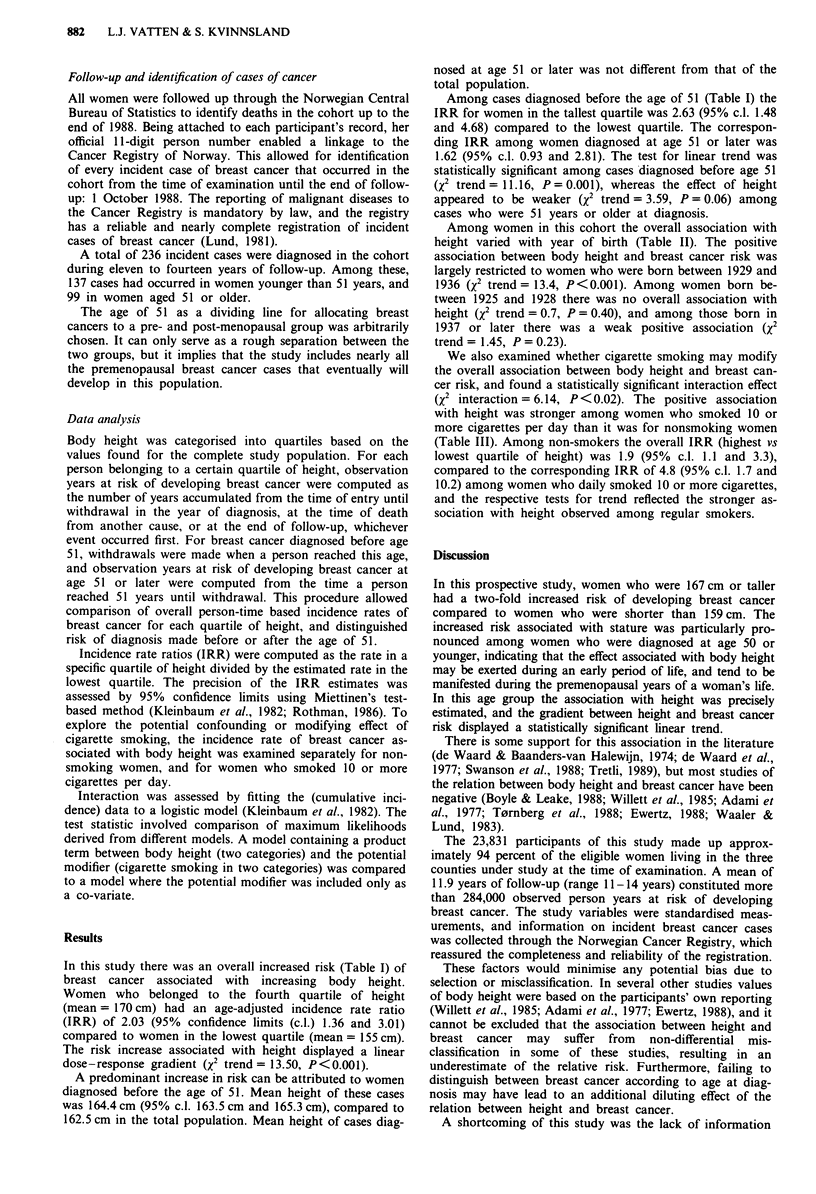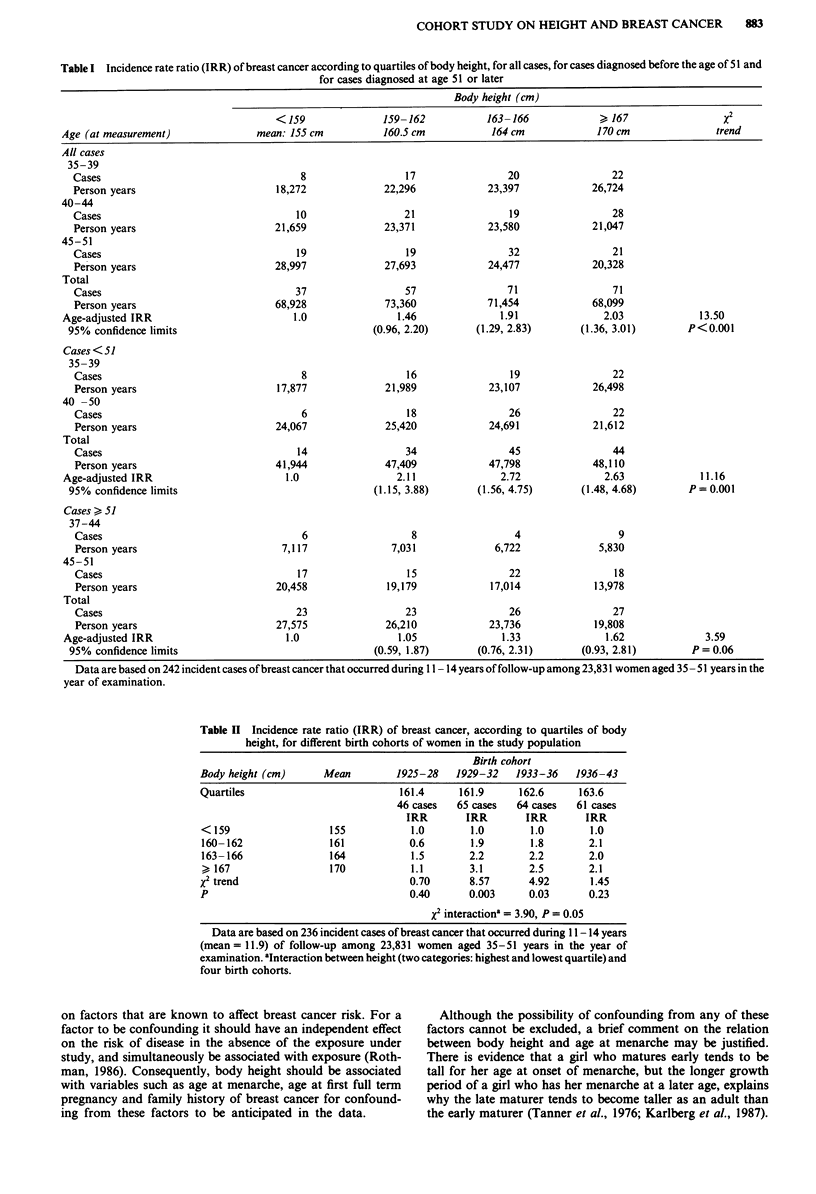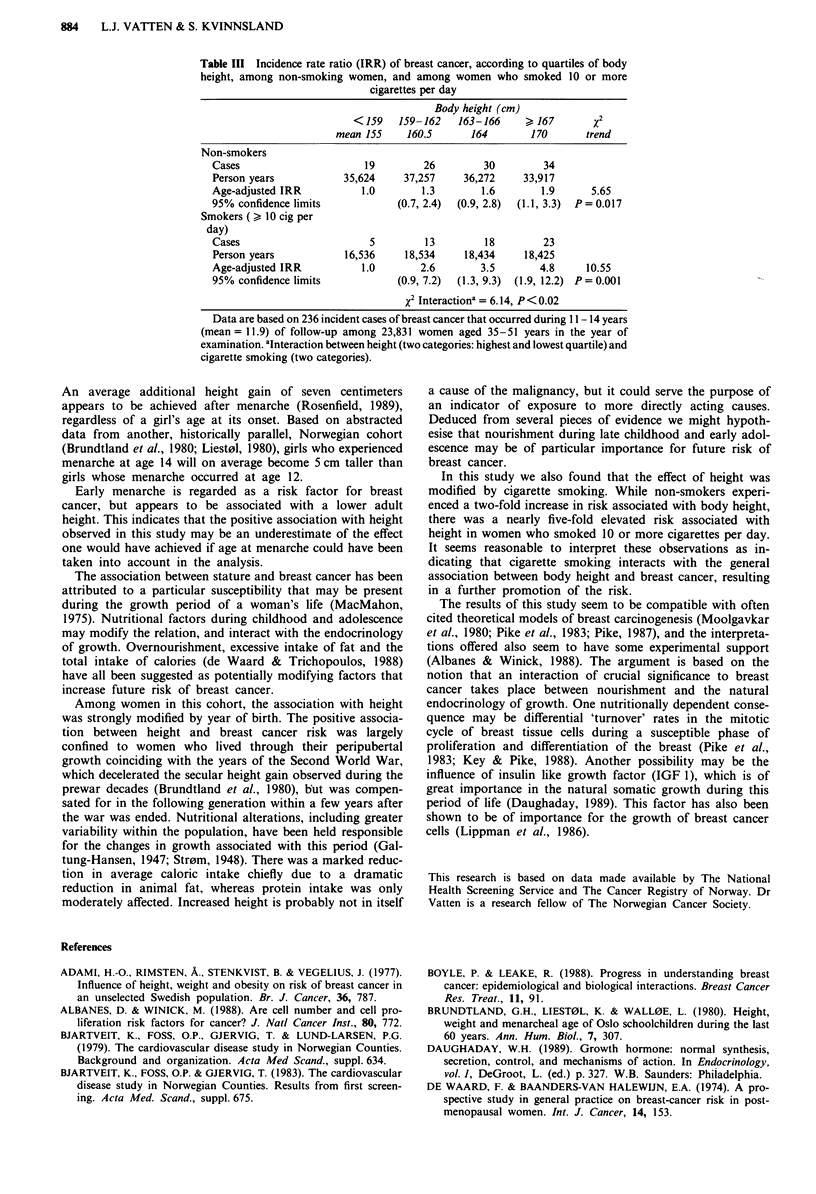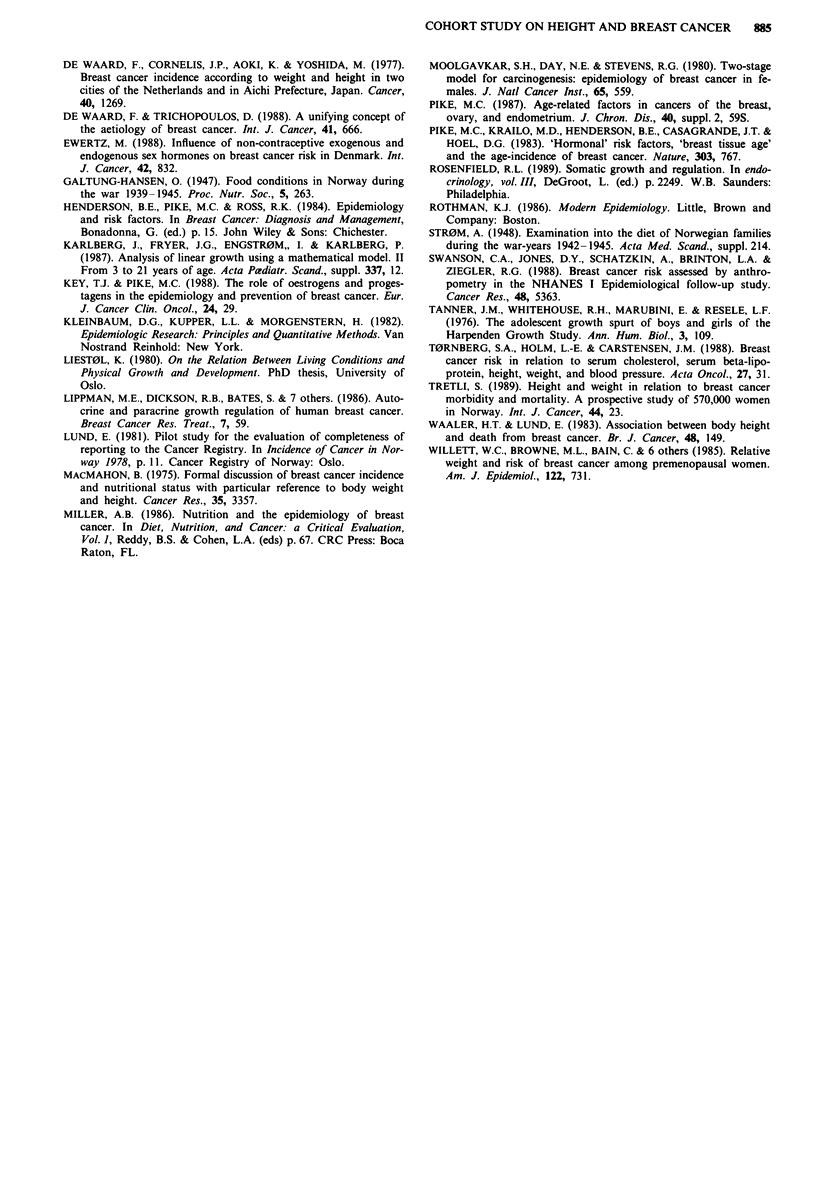# Body height and risk of breast cancer. A prospective study of 23,831 Norwegian women.

**DOI:** 10.1038/bjc.1990.197

**Published:** 1990-06

**Authors:** L. J. Vatten, S. Kvinnsland

**Affiliations:** Department of Oncology, University Hospital, Trondheim, Norway.

## Abstract

The association between body height and the incidence rate of breast cancer has been examined in 236 cases of breast cancer that occurred among 23,831 Norwegian women during 11-14 years of follow-up. At the time of height measurement they were 35-51 years of age. The age-adjusted incidence rate ratio (IRR) of breast cancer was 2.03 (95% of confidence limits 1.36 and 3.01) for women taller than or equal to 167 cm (mean = 170 cm) compared to women who were less than 159 cm (mean = 155 cm). The positive association with height was stronger among women who were diagnosed before the age of 51 (IRR = 2.63; 95% confidence limits 1.48 and 4.68), than among women diagnosed after this age. Moreover, the association appeared to be confined to women who had lived through their peripubertal growth during a period (1940-45) of nationally increased nutritional variability with reduction in dietary fat and restricted caloric intake. Among women born between 1929 and 1936, the relation with height displayed a strong positive linear trend (chi 2 trend = 13.4, P less than 0.001), which was not present among women born between 1925 and 1928 (chi 2 trend = 0.7, P = 0.40), nor among women born in 1937 or later (chi 2 trend = 1.5, P = 0.20). We hypothesise that a time-dependent diversity in nourishment, which may be of particular importance for women in their peri-menarcheal development, may explain the different association between body height and breast cancer risk that was observed for women in different birth cohorts.


*Br. J. Cancer* (1990), **61**, 881–885 © Macmillan Press Ltd., 1990

### Body height and risk of breast cancer. A prospective study of 23,831 Norwegian women

L.J. Vatten[1,2,3] & S. Kvinnsland[1]

[1]*Department of Oncology, University Hospital, N-7006 Trondheim, Norway;* [2]*The Norwegian Cancer Registry, Montebello, N-0310 Oslo 3, Norway; in collaboration with* [3]*The National Health Screening Service, PO Box 8155 Dep., Oslo 1, Norway.*

**Summary** The association between body height and the incidence rate of breast cancer has been examined in 236 cases of breast cancer that occurred among 23,831 Norwegian women during 11–14 years of follow-up. At the time of height measurement they were 35–51 years of age. The age-adjusted incidence rate ratio (IRR) of breast cancer was 2.03 (95% of confidence limits 1.36 and 3.01) for women taller than or equal to 167 cm (mean = 170 cm) compared to women who were less than 159 cm (mean = 155 cm). The positive association with height was stronger among women who were diagnosed before the age of 51 (IRR = 2.63; 95% confidence limits 1.48 and 4.68), than among women diagnosed after this age. Moreover, the association appeared to be confined to women who had lived through their peripubertal growth during a period (1940–45) of nationally increased nutritional variability with reduction in dietary fat and restricted caloric intake. Among women born between 1929 and 1936, the relation with height displayed a strong positive linear trend ($\chi^2$ trend = 13.4, $P<0.001$), which was not present among women born between 1925 and 1928 ($\chi^2$ trend = 0.7, $P = 0.40$), nor among women born in 1937 or later ($\chi^2$ trend = 1.5, $P = 0.20$). We hypothesise that a time-dependent diversity in nourishment, which may be of particular importance for women in their peri-menarcheal development, may explain the different association between body height and breast cancer risk that was observed for women in different birth cohorts.

The associations between anthropometric indices and risk of breast cancer have often been interpreted as nutritionally dependent relations. Whereas body weight appears to be an established risk factor for breast cancer in post-menopausal women (Henderson *et al.*, 1984; Boyle & Leake, 1988), studies on the effect of height have mostly been negative (Willett *et al.*, 1985; Adami *et al.*, 1977; Tørnberg *et al.*, 1988; Ewertz, 1988; Waaler & Lund, 1983). Some studies, however, have found body height to be positively associated with the risk of breast cancer (de Waard & Baanders-van Halewijn, 1974; de Waard *et al.*, 1977; Swanson *et al.*, 1988).

One population-based case–control study (de Waard *et al.*, 1977) compared the incidence of breast cancer in a Dutch population with that of a Japanese, and concluded that 51% of the incidence difference could be attributed to the greater height in the Dutch population. A relatively large prospective study (Swanson *et al.*, 1988) reported 90% increased risk of breast cancer among women in the highest quintile of height compared to those in the lowest quintile.

If an unbiased association were to exist between body height and risk of breast cancer, it would be warranted to pay closer attention to the peripubertal and adolescent period in a woman's life (MacMahon, 1975; Miller, 1986), during which her body height is determined, and her breast tissue is being developed. Environmental conditions, such as nutritional influences during childhood and adolescence (de Waard & Trichopoulos, 1988), may be of importance for later development of breast cancer. Biologically, understanding the endocrinology of natural growth and development during this period may provide further insight into the pathogenesis of breast cancer.

We have studied a cohort of women, a substantial proportion of whom lived through their pubertal years during a period of national food restriction (1940–45), which reduced the secular height gain in the population as a whole (Brundtland *et al.*, 1980). If the relation between height and breast cancer was different among women whose menarche occurred during this period, compared to the association with height among women who had their pubertal growth before or after this time, nutritional hypotheses related to a susceptible phase of breast tissue development may be warranted for further understanding the aetiology of breast cancer.

Correspondence: L.J. Vatten, Department of Oncology, University Hospital, N-7006 Trondheim, Norway.
Received 3 July 1989; and in revised form 14 December 1989.

### Methods

### *The study population*

From 1974 to 1977 all men and women aged 35–49 years living in three separate counties in Norway were invited to participate in a health screening examination organised by the National Health Screening Service. The screening procedure included a questionnaire and standardised measurements of height, weight, and blood pressure. A chest X-ray was taken, and a non-fasting blood sample was drawn and analysed on total serum cholesterol, triglycerides, and glucose. The residual blood sera have since been kept frozen in a special serum bank.

A comprehensive description of the health screening procedures has previously been given by Bjartveit *et al.* (1979, 1983).

A total of 26,252 women were invited, among whom 24,617 (93.8%) attended the examination. Measurement of height was missing in 499 women, and these were excluded from analysis. A majority of the missing height measurements were found in pregnant women. This can be explained by the fact that height and weight measurements were performed in conjunction with the X-ray procedure, from which pregnant women were exempted. Moreover, all cancer cases (including breast cancer) that had occurred before or during the calendar year of examination were excluded. Altogether, these counted 287 women, thus leaving a total of 23,831 women eligible for analysis.

### *The questionnaire*

The main objective of the primary questionnaire was to obtain information on known and suspected risk factors for cardiovascular disease. Consequently, there was a lack of information on factors that are known to predict breast cancer, such as age at menarche, age at first full term pregnancy, family history of breast cancer, and reliable information on exact age at menopause. However, the questionnaire did include detailed history of past and current smoking habits, and various demographic variables.

*Follow-up and identification of cases of cancer*

All women were followed up through the Norwegian Central Bureau of Statistics to identify deaths in the cohort up to the end of 1988. Being attached to each participant's record, her official 11-digit person number enabled a linkage to the Cancer Registry of Norway. This allowed for identification of every incident case of breast cancer that occurred in the cohort from the time of examination until the end of follow-up: 1 October 1988. The reporting of malignant diseases to the Cancer Registry is mandatory by law, and the registry has a reliable and nearly complete registration of incident cases of breast cancer (Lund, 1981).

A total of 236 incident cases were diagnosed in the cohort during eleven to fourteen years of follow-up. Among these, 137 cases had occurred in women younger than 51 years, and 99 in women aged 51 or older.

The age of 51 as a dividing line for allocating breast cancers to a pre- and post-menopausal group was arbitrarily chosen. It can only serve as a rough separation between the two groups, but it implies that the study includes nearly all the premenopausal breast cancer cases that eventually will develop in this population.

*Data analysis*

Body height was categorised into quartiles based on the values found for the complete study population. For each person belonging to a certain quartile of height, observation years at risk of developing breast cancer were computed as the number of years accumulated from the time of entry until withdrawal in the year of diagnosis, at the time of death from another cause, or at the end of follow-up, whichever event occurred first. For breast cancer diagnosed before age 51, withdrawals were made when a person reached this age, and observation years at risk of developing breast cancer at age 51 or later were computed from the time a person reached 51 years until withdrawal. This procedure allowed comparison of overall person-time based incidence rates of breast cancer for each quartile of height, and distinguished risk of diagnosis made before or after the age of 51.

Incidence rate ratios (IRR) were computed as the rate in a specific quartile of height divided by the estimated rate in the lowest quartile. The precision of the IRR estimates was assessed by 95% confidence limits using Miettinen's test-based method (Kleinbaum *et al.*, 1982; Rothman, 1986). To explore the potential confounding or modifying effect of cigarette smoking, the incidence rate of breast cancer associated with body height was examined separately for non-smoking women, and for women who smoked 10 or more cigarettes per day.

Interaction was assessed by fitting the (cumulative incidence) data to a logistic model (Kleinbaum *et al.*, 1982). The test statistic involved comparison of maximum likelihoods derived from different models. A model containing a product term between body height (two categories) and the potential modifier (cigarette smoking in two categories) was compared to a model where the potential modifier was included only as a co-variate.

### Results

In this study there was an overall increased risk (Table I) of breast cancer associated with increasing body height. Women who belonged to the fourth quartile of height (mean = 170 cm) had an age-adjusted incidence rate ratio (IRR) of 2.03 (95% confidence limits (c.l.) 1.36 and 3.01) compared to women in the lowest quartile (mean = 155 cm). The risk increase associated with height displayed a linear dose–response gradient ($\chi^2$ trend = 13.50, $P<0.001$).

A predominant increase in risk can be attributed to women diagnosed before the age of 51. Mean height of these cases was 164.4 cm (95% c.l. 163.5 cm and 165.3 cm), compared to 162.5 cm in the total population. Mean height of cases diagnosed at age 51 or later was not different from that of the total population.

Among cases diagnosed before the age of 51 (Table I) the IRR for women in the tallest quartile was 2.63 (95% c.l. 1.48 and 4.68) compared to the lowest quartile. The corresponding IRR among women diagnosed at age 51 or later was 1.62 (95% c.l. 0.93 and 2.81). The test for linear trend was statistically significant among cases diagnosed before age 51 ($\chi^2$ trend = 11.16, $P = 0.001$), whereas the effect of height appeared to be weaker ($\chi^2$ trend = 3.59, $P = 0.06$) among cases who were 51 years or older at diagnosis.

Among women in this cohort the overall association with height varied with year of birth (Table II). The positive association between body height and breast cancer risk was largely restricted to women who were born between 1929 and 1936 ($\chi^2$ trend = 13.4, $P<0.001$). Among women born between 1925 and 1928 there was no overall association with height ($\chi^2$ trend = 0.7, $P = 0.40$), and among those born in 1937 or later there was a weak positive association ($\chi^2$ trend = 1.45, $P = 0.23$).

We also examined whether cigarette smoking may modify the overall association between body height and breast cancer risk, and found a statistically significant interaction effect ($\chi^2$ interaction = 6.14, $P<0.02$). The positive association with height was stronger among women who smoked 10 or more cigarettes per day than it was for nonsmoking women (Table III). Among non-smokers the overall IRR (highest *vs* lowest quartile of height) was 1.9 (95% c.l. 1.1 and 3.3), compared to the corresponding IRR of 4.8 (95% c.l. 1.7 and 10.2) among women who daily smoked 10 or more cigarettes, and the respective tests for trend reflected the stronger association with height observed among regular smokers.

### Discussion

In this prospective study, women who were 167 cm or taller had a two-fold increased risk of developing breast cancer compared to women who were shorter than 159 cm. The increased risk associated with stature was particularly pronounced among women who were diagnosed at age 50 or younger, indicating that the effect associated with body height may be exerted during an early period of life, and tend to be manifested during the premenopausal years of a woman's life. In this age group the association with height was precisely estimated, and the gradient between height and breast cancer risk displayed a statistically significant linear trend.

There is some support for this association in the literature (de Waard & Baanders-van Halewijn, 1974; de Waard *et al.*, 1977; Swanson *et al.*, 1988; Tretli, 1989), but most studies of the relation between body height and breast cancer have been negative (Boyle & Leake, 1988; Willett *et al.*, 1985; Adami *et al.*, 1977; Tørnberg *et al.*, 1988; Ewertz, 1988; Waaler & Lund, 1983).

The 23,831 participants of this study made up approximately 94 percent of the eligible women living in the three counties under study at the time of examination. A mean of 11.9 years of follow-up (range 11–14 years) constituted more than 284,000 observed person years at risk of developing breast cancer. The study variables were standardised measurements, and information on incident breast cancer cases was collected through the Norwegian Cancer Registry, which reassured the completeness and reliability of the registration.

These factors would minimise any potential bias due to selection or misclassification. In several other studies values of body height were based on the participants' own reporting (Willett *et al.*, 1985; Adami *et al.*, 1977; Ewertz, 1988), and it cannot be excluded that the association between height and breast cancer may suffer from non-differential misclassification in some of these studies, resulting in an underestimate of the relative risk. Furthermore, failing to distinguish between breast cancer according to age at diagnosis may have lead to an additional diluting effect of the relation between height and breast cancer.

A shortcoming of this study was the lack of information

**Table I** Incidence rate ratio (IRR) of breast cancer according to quartiles of body height, for all cases, for cases diagnosed before the age of 51 and for cases diagnosed at age 51 or later

| | *Body height (cm)* | | | | |
|---|---|---|---|---|---|
| *Age (at measurement)* | *< 159* *mean: 155 cm* | *159–162* *160.5 cm* | *163–166* *164 cm* | *≥ 167* *170 cm* | $\chi^2$ *trend* |
| *All cases* | | | | | |
| 35–39 | | | | | |
| Cases | 8 | 17 | 20 | 22 | |
| Person years | 18,272 | 22,296 | 23,397 | 26,724 | |
| 40–44 | | | | | |
| Cases | 10 | 21 | 19 | 28 | |
| Person years | 21,659 | 23,371 | 23,580 | 21,047 | |
| 45–51 | | | | | |
| Cases | 19 | 19 | 32 | 21 | |
| Person years | 28,997 | 27,693 | 24,477 | 20,328 | |
| Total | | | | | |
| Cases | 37 | 57 | 71 | 71 | |
| Person years | 68,928 | 73,360 | 71,454 | 68,099 | |
| Age-adjusted IRR | 1.0 | 1.46 | 1.91 | 2.03 | 13.50 |
| 95% confidence limits | | (0.96, 2.20) | (1.29, 2.83) | (1.36, 3.01) | $P<0.001$ |
| *Cases < 51* | | | | | |
| 35–39 | | | | | |
| Cases | 8 | 16 | 19 | 22 | |
| Person years | 17,877 | 21,989 | 23,107 | 26,498 | |
| 40 –50 | | | | | |
| Cases | 6 | 18 | 26 | 22 | |
| Person years | 24,067 | 25,420 | 24,691 | 21,612 | |
| Total | | | | | |
| Cases | 14 | 34 | 45 | 44 | |
| Person years | 41,944 | 47,409 | 47,798 | 48,110 | |
| Age-adjusted IRR | 1.0 | 2.11 | 2.72 | 2.63 | 11.16 |
| 95% confidence limits | | (1.15, 3.88) | (1.56, 4.75) | (1.48, 4.68) | $P = 0.001$ |
| *Cases ≥ 51* | | | | | |
| 37–44 | | | | | |
| Cases | 6 | 8 | 4 | 9 | |
| Person years | 7,117 | 7,031 | 6,722 | 5,830 | |
| 45–51 | | | | | |
| Cases | 17 | 15 | 22 | 18 | |
| Person years | 20,458 | 19,179 | 17,014 | 13,978 | |
| Total | | | | | |
| Cases | 23 | 23 | 26 | 27 | |
| Person years | 27,575 | 26,210 | 23,736 | 19,808 | |
| Age-adjusted IRR | 1.0 | 1.05 | 1.33 | 1.62 | 3.59 |
| 95% confidence limits | | (0.59, 1.87) | (0.76, 2.31) | (0.93, 2.81) | $P = 0.06$ |

Data are based on 242 incident cases of breast cancer that occurred during 11–14 years of follow-up among 23,831 women aged 35–51 years in the year of examination.

**Table II** Incidence rate ratio (IRR) of breast cancer, according to quartiles of body height, for different birth cohorts of women in the study population

| | | *Birth cohort* | | | |
|---|---|---|---|---|---|
| *Body height (cm)* | *Mean* | *1925–28* | *1929–32* | *1933–36* | *1936–43* |
| Quartiles | | 161.4 | 161.9 | 162.6 | 163.6 |
| | | 46 cases | 65 cases | 64 cases | 61 cases |
| | | IRR | IRR | IRR | IRR |
| < 159 | 155 | 1.0 | 1.0 | 1.0 | 1.0 |
| 160–162 | 161 | 0.6 | 1.9 | 1.8 | 2.1 |
| 163–166 | 164 | 1.5 | 2.2 | 2.2 | 2.0 |
| ≥ 167 | 170 | 1.1 | 3.1 | 2.5 | 2.1 |
| $\chi^2$ trend | | 0.70 | 8.57 | 4.92 | 1.45 |
| *P* | | 0.40 | 0.003 | 0.03 | 0.23 |
| | | $\chi^2$ interaction[a] = 3.90, $P = 0.05$ | | | |

Data are based on 236 incident cases of breast cancer that occurred during 11–14 years (mean = 11.9) of follow-up among 23,831 women aged 35–51 years in the year of examination. [a]Interaction between height (two categories: highest and lowest quartile) and four birth cohorts.

on factors that are known to affect breast cancer risk. For a factor to be confounding it should have an independent effect on the risk of disease in the absence of the exposure under study, and simultaneously be associated with exposure (Rothman, 1986). Consequently, body height should be associated with variables such as age at menarche, age at first full term pregnancy and family history of breast cancer for confounding from these factors to be anticipated in the data.

Although the possibility of confounding from any of these factors cannot be excluded, a brief comment on the relation between body height and age at menarche may be justified. There is evidence that a girl who matures early tends to be tall for her age at onset of menarche, but the longer growth period of a girl who has her menarche at a later age, explains why the late maturer tends to become taller as an adult than the early maturer (Tanner *et al.*, 1976; Karlberg *et al.*, 1987).

**Table III** Incidence rate ratio (IRR) of breast cancer, according to quartiles of body height, among non-smoking women, and among women who smoked 10 or more cigarettes per day

| | *Body height (cm)* | | | | |
|---|---|---|---|---|---|
| | *<159 mean 155* | *159–162 160.5* | *163–166 164* | *≥167 170* | $\chi^2$ *trend* |
| Non-smokers | | | | | |
| Cases | 19 | 26 | 30 | 34 | |
| Person years | 35,624 | 37,257 | 36,272 | 33,917 | |
| Age-adjusted IRR | 1.0 | 1.3 | 1.6 | 1.9 | 5.65 |
| 95% confidence limits | | (0.7, 2.4) | (0.9, 2.8) | (1.1, 3.3) | $P = 0.017$ |
| Smokers (≥10 cig per day) | | | | | |
| Cases | 5 | 13 | 18 | 23 | |
| Person years | 16,536 | 18,534 | 18,434 | 18,425 | |
| Age-adjusted IRR | 1.0 | 2.6 | 3.5 | 4.8 | 10.55 |
| 95% confidence limits | | (0.9, 7.2) | (1.3, 9.3) | (1.9, 12.2) | $P = 0.001$ |
| | | $\chi^2$ Interaction[a] = 6.14, $P < 0.02$ | | | |

Data are based on 236 incident cases of breast cancer that occurred during 11–14 years (mean = 11.9) of follow-up among 23,831 women aged 35–51 years in the year of examination. [a]Interaction between height (two categories: highest and lowest quartile) and cigarette smoking (two categories).

An average additional height gain of seven centimeters appears to be achieved after menarche (Rosenfield, 1989), regardless of a girl's age at its onset. Based on abstracted data from another, historically parallel, Norwegian cohort (Brundtland *et al.*, 1980; Liestøl, 1980), girls who experienced menarche at age 14 will on average become 5 cm taller than girls whose menarche occurred at age 12.

Early menarche is regarded as a risk factor for breast cancer, but appears to be associated with a lower adult height. This indicates that the positive association with height observed in this study may be an underestimate of the effect one would have achieved if age at menarche could have been taken into account in the analysis.

The association between stature and breast cancer has been attributed to a particular susceptibility that may be present during the growth period of a woman's life (MacMahon, 1975). Nutritional factors during childhood and adolescence may modify the relation, and interact with the endocrinology of growth. Overnourishment, excessive intake of fat and the total intake of calories (de Waard & Trichopoulos, 1988) have all been suggested as potentially modifying factors that increase future risk of breast cancer.

Among women in this cohort, the association with height was strongly modified by year of birth. The positive association between height and breast cancer risk was largely confined to women who lived through their peripubertal growth coinciding with the years of the Second World War, which decelerated the secular height gain observed during the prewar decades (Brundtland *et al.*, 1980), but was compensated for in the following generation within a few years after the war was ended. Nutritional alterations, including greater variability within the population, have been held responsible for the changes in growth associated with this period (Galtung-Hansen, 1947; Strøm, 1948). There was a marked reduction in average caloric intake chiefly due to a dramatic reduction in animal fat, whereas protein intake was only moderately affected. Increased height is probably not in itself a cause of the malignancy, but it could serve the purpose of an indicator of exposure to more directly acting causes. Deduced from several pieces of evidence we might hypothesise that nourishment during late childhood and early adolescence may be of particular importance for future risk of breast cancer.

In this study we also found that the effect of height was modified by cigarette smoking. While non-smokers experienced a two-fold increase in risk associated with body height, there was a nearly five-fold elevated risk associated with height in women who smoked 10 or more cigarettes per day. It seems reasonable to interpret these observations as indicating that cigarette smoking interacts with the general association between body height and breast cancer, resulting in a further promotion of the risk.

The results of this study seem to be compatible with often cited theoretical models of breast carcinogenesis (Moolgavkar *et al.*, 1980; Pike *et al.*, 1983; Pike, 1987), and the interpretations offered also seem to have some experimental support (Albanes & Winick, 1988). The argument is based on the notion that an interaction of crucial significance to breast cancer takes place between nourishment and the natural endocrinology of growth. One nutritionally dependent consequence may be differential 'turnover' rates in the mitotic cycle of breast tissue cells during a susceptible phase of proliferation and differentiation of the breast (Pike *et al.*, 1983; Key & Pike, 1988). Another possibility may be the influence of insulin like growth factor (IGF 1), which is of great importance in the natural somatic growth during this period of life (Daughaday, 1989). This factor has also been shown to be of importance for the growth of breast cancer cells (Lippman *et al.*, 1986).

This research is based on data made available by The National Health Screening Service and The Cancer Registry of Norway. Dr Vatten is a research fellow of The Norwegian Cancer Society.

### References

ADAMI, H.-O., RIMSTEN, Å., STENKVIST, B. & VEGELIUS, J. (1977). Influence of height, weight and obesity on risk of breast cancer in an unselected Swedish population. *Br. J. Cancer*, **36**, 787.

ALBANES, D. & WINICK, M. (1988). Are cell number and cell proliferation risk factors for cancer? *J. Natl Cancer Inst.*, **80**, 772.

BJARTVEIT, K., FOSS, O.P., GJERVIG, T. & LUND-LARSEN, P.G. (1979). The cardiovascular disease study in Norwegian Counties. Background and organization. *Acta Med Scand.*, suppl. 634.

BJARTVEIT, K., FOSS, O.P. & GJERVIG, T. (1983). The cardiovascular disease study in Norwegian Counties. Results from first screening. *Acta Med. Scand.*, suppl. 675.

BOYLE, P. & LEAKE, R. (1988). Progress in understanding breast cancer: epidemiological and biological interactions. *Breast Cancer Res. Treat.*, **11**, 91.

BRUNDTLAND, G.H., LIESTØL, K. & WALLØE, L. (1980). Height, weight and menarcheal age of Oslo schoolchildren during the last 60 years. *Ann. Hum. Biol.*, **7**, 307.

DAUGHADAY, W.H. (1989). Growth hormone: normal synthesis, secretion, control, and mechanisms of action. In *Endocrinology, vol. 1*, DeGroot, L. (ed.) p. 327. W.B. Saunders: Philadelphia.

DE WAARD, F. & BAANDERS-VAN HALEWIJN, E.A. (1974). A prospective study in general practice on breast-cancer risk in postmenopausal women. *Int. J. Cancer*, **14**, 153.

DE WAARD, F., CORNELIS, J.P., AOKI, K. & YOSHIDA, M. (1977). Breast cancer incidence according to weight and height in two cities of the Netherlands and in Aichi Prefecture, Japan. *Cancer*, **40**, 1269.

DE WAARD, F. & TRICHOPOULOS, D. (1988). A unifying concept of the aetiology of breast cancer. *Int. J. Cancer*, **41**, 666.

EWERTZ, M. (1988). Influence of non-contraceptive exogenous and endogenous sex hormones on breast cancer risk in Denmark. *Int. J. Cancer*, **42**, 832.

GALTUNG-HANSEN, O. (1947). Food conditions in Norway during the war 1939–1945. *Proc. Nutr. Soc.*, **5**, 263.

HENDERSON, B.E., PIKE, M.C. & ROSS, R.K. (1984). Epidemiology and risk factors. In *Breast Cancer: Diagnosis and Management*, Bonadonna, G. (ed.) p. 15. John Wiley & Sons: Chichester.

KARLBERG, J., FRYER, J.G., ENGSTRØM,, I. & KARLBERG, P. (1987). Analysis of linear growth using a mathematical model. II From 3 to 21 years of age. *Acta Pædiatr. Scand.*, suppl. **337**, 12.

KEY, T.J. & PIKE, M.C. (1988). The role of oestrogens and progestagens in the epidemiology and prevention of breast cancer. *Eur. J. Cancer Clin. Oncol.*, **24**, 29.

KLEINBAUM, D.G., KUPPER, L.L. & MORGENSTERN, H. (1982). *Epidemiologic Research: Principles and Quantitative Methods*. Van Nostrand Reinhold: New York.

LIESTØL, K. (1980). *On the Relation Between Living Conditions and Physical Growth and Development*. PhD thesis, University of Oslo.

LIPPMAN, M.E., DICKSON, R.B., BATES, S. & 7 others. (1986). Autocrine and paracrine growth regulation of human breast cancer. *Breast Cancer Res. Treat.*, **7**, 59.

LUND, E. (1981). Pilot study for the evaluation of completeness of reporting to the Cancer Registry. In *Incidence of Cancer in Norway 1978*, p. 11. Cancer Registry of Norway: Oslo.

MACMAHON, B. (1975). Formal discussion of breast cancer incidence and nutritional status with particular reference to body weight and height. *Cancer Res.*, **35**, 3357.

MILLER, A.B. (1986). Nutrition and the epidemiology of breast cancer. In *Diet, Nutrition, and Cancer: a Critical Evaluation, Vol. 1*, Reddy, B.S. & Cohen, L.A. (eds) p. 67. CRC Press: Boca Raton, FL.

MOOLGAVKAR, S.H., DAY, N.E. & STEVENS, R.G. (1980). Two-stage model for carcinogenesis: epidemiology of breast cancer in females. *J. Natl Cancer Inst.*, **65**, 559.

PIKE, M.C. (1987). Age-related factors in cancers of the breast, ovary, and endometrium. *J. Chron. Dis.*, **40**, suppl. 2, 59S.

PIKE, M.C., KRAILO, M.D., HENDERSON, B.E., CASAGRANDE, J.T. & HOEL, D.G. (1983). 'Hormonal' risk factors, 'breast tissue age' and the age-incidence of breast cancer. *Nature*, **303**, 767.

ROSENFIELD, R.L. (1989). Somatic growth and regulation. In *endocrinology, vol. III*, DeGroot, L. (ed.) p. 2249. W.B. Saunders: Philadelphia.

ROTHMAN, K.J. (1986). *Modern Epidemiology*. Little, Brown and Company: Boston.

STRØM, A. (1948). Examination into the diet of Norwegian families during the war-years 1942–1945. *Acta Med. Scand.*, suppl. 214.

SWANSON, C.A., JONES, D.Y., SCHATZKIN, A., BRINTON, L.A. & ZIEGLER, R.G. (1988). Breast cancer risk assessed by anthropometry in the NHANES I Epidemiological follow-up study. *Cancer Res.*, **48**, 5363.

TANNER, J.M., WHITEHOUSE, R.H., MARUBINI, E. & RESELE, L.F. (1976). The adolescent growth spurt of boys and girls of the Harpenden Growth Study. *Ann. Hum. Biol.*, **3**, 109.

TØRNBERG, S.A., HOLM, L.-E. & CARSTENSEN, J.M. (1988). Breast cancer risk in relation to serum cholesterol, serum beta-lipoprotein, height, weight, and blood pressure. *Acta Oncol.*, **27**, 31.

TRETLI, S. (1989). Height and weight in relation to breast cancer morbidity and mortality. A prospective study of 570,000 women in Norway. *Int. J. Cancer*, **44**, 23.

WAALER, H.T. & LUND, E. (1983). Association between body height and death from breast cancer. *Br. J. Cancer*, **48**, 149.

WILLETT, W.C., BROWNE, M.L., BAIN, C. & 6 others (1985). Relative weight and risk of breast cancer among premenopausal women. *Am. J. Epidemiol.*, **122**, 731.